# Presentations to Emergency Departments for COPD: A Time Series Analysis

**DOI:** 10.1155/2016/1382434

**Published:** 2016-04-04

**Authors:** Rhonda J. Rosychuk, Erik Youngson, Brian H. Rowe

**Affiliations:** ^1^Department of Pediatrics, University of Alberta, Edmonton, AB, Canada T6G 1C9; ^2^Patient Health Outcomes Research and Clinical Effectiveness Unit, University of Alberta, Edmonton, AB, Canada T6G 2M8; ^3^Department of Emergency Medicine, University of Alberta, Edmonton, AB, Canada T6G 2R7; ^4^School of Public Health, University of Alberta, Edmonton, AB, Canada T6G 1C9; ^5^Alberta Health Services, Edmonton, AB, Canada T5J 3E4

## Abstract

*Background*. Chronic obstructive pulmonary disease (COPD) is a common respiratory condition characterized by progressive dyspnea and acute exacerbations which may result in emergency department (ED) presentations. This study examines monthly rates of presentations to EDs in one Canadian province.* Methods*. Presentations for COPD made by individuals aged ≥55 years during April 1999 to March 2011 were extracted from provincial databases. Data included age, sex, and health zone of residence (North, Central, South, and urban). Crude rates were calculated. Seasonal autoregressive integrated moving average (SARIMA) time series models were developed.* Results*. ED presentations for COPD totalled 188,824 and the monthly rate of presentation remained relatively stable (from 197.7 to 232.6 per 100,000). Males and seniors (≥65 years) comprised 52.2% and 73.7% of presentations, respectively. The ARIMA(1,0, 0) × (1,0, 1)_12_ model was appropriate for the overall rate of presentations and for each sex and seniors. Zone specific models showed relatively stable or decreasing rates; the North zone had an increasing trend.* Conclusions*. ED presentation rates for COPD have been relatively stable in Alberta during the past decade. However, their increases in northern regions deserve further exploration. The SARIMA models quantified the temporal patterns and can help planning future health care service needs.

## 1. Introduction

Chronic obstructive pulmonary disease (COPD) is a common and disabling condition usually caused by cigarette smoke exposure and characterized by progressive symptoms of dyspnea. Acute exacerbations of COPD (AECOPD) are characterized by an increase in the ongoing symptoms of cough, sputum production, sputum purulence, and/or shortness of breath which usually require changes in baseline medical management [1, 2]. Overall, these COPD exacerbations vary in severity from mild to life threatening and are expected to be the third leading cause of death worldwide by 2030 [3].

Patients with COPD often experience exacerbations and some exacerbations are severe enough to require emergency department (ED) management. Treatment guidelines for AECOPD exist and prolonged treatment may occur in the ED, some exacerbations result in complications including pneumothorax, pneumonia, need for noninvasive ventilation, intubation and mechanical ventilation, and death. Due to the advanced age of patients, associated severe comorbidities, and severity of many presentations, hospital admission is a common outcome in severe exacerbations (33% in COPD [4] versus 9% in asthma [5]). Not surprisingly, among ambulatory care sensitive conditions, COPD is the leading reason for hospitalizations in Canada [3].

Based on data health professional-diagnosed COPD from the 1994-95 National Health Survey, prevalence rates are estimated to be 4.7%, 5.4%, and 8.3% in Canadians aged 55–64, 65–74, and ≥75, respectively [6]. More recently, 2012 national data from the Canadian Primary Care Sentinel Surveillance Network provide primary care rates of diagnosed COPD of 3.3%, 6.6%, 11.3%, and 14.8% for ages 50–59, 60–69, 70–79, and 80+, respectively, while trends suggest that disease prevalence and first listed hospital discharge rate is stable in men; however, the prevalence is increasing among women, reflecting a 40-year societal trend towards increased smoking among females [7].

ED presentations for COPD are important events for patients and the health care system. This study describes the temporal trends in the presentations of individuals (age ≥55 years) to EDs for COPD during a 12-year period (April 1, 1999, to March 31, 2011) and provides time series models to quantify the temporal patterns.

## 2. Methods

### 2.1. Study Design

This project is a secondary analysis of existing population-based, administrative databases.

### 2.2. Study Setting and Population

Alberta is a Western Canadian province, which operates under a single health authority, providing government funded health care services to more than 4.1 million residents. All visits to Alberta's more than 100 EDs are tracked in the provincial Ambulatory Care Classification System [8] (ACCS). This system has transitioned to the National Ambulatory Care Reporting System [9] (NACRS). The administrative database contains pertinent data about ED visits including start and end dates and times, age, acuity, disposition status, and diagnostic fields including the main diagnosis and up to nine additional diagnoses (only five prior to April 1, 2002), as recorded by trained health record nosologists using the International Classification of Diseases, Ninth Revision, Clinical Modification [10] (ICD-9-CM; prior to April 1, 2002) or International Classification of Diseases, Tenth Revision, Canadian Enhancement [11] (ICD-10-CA; since April 1, 2002). Linkage with the Alberta Health Care Insurance Plan (AHCIP; provincial registry) provides demographic data including age, sex, and geographic area of residence at the end of the fiscal year for all patients. The AHCIP also provides annual population counts by age, sex, and geographic area of residence.

### 2.3. Study Protocol

All COPD ED visits for patients 55 years or older between April 1, 1999, and March 31, 2011 (fiscal years 2000 to 2011), were extracted from ACCS, where COPD was defined by either the first or second diagnosis field containing any of the diagnostic codes 490.x, 491.x, 492.x, 494.x, and 496.x (ICD-9-CM) or J40.x, J41.x, J42.x, J43.x, J44.x, and J47.x (ICD-10-CA). The date and time of the ED visit were extracted as well as the mode of release from the ED (i.e., admitted to hospital or discharged).

**Figure 1:**
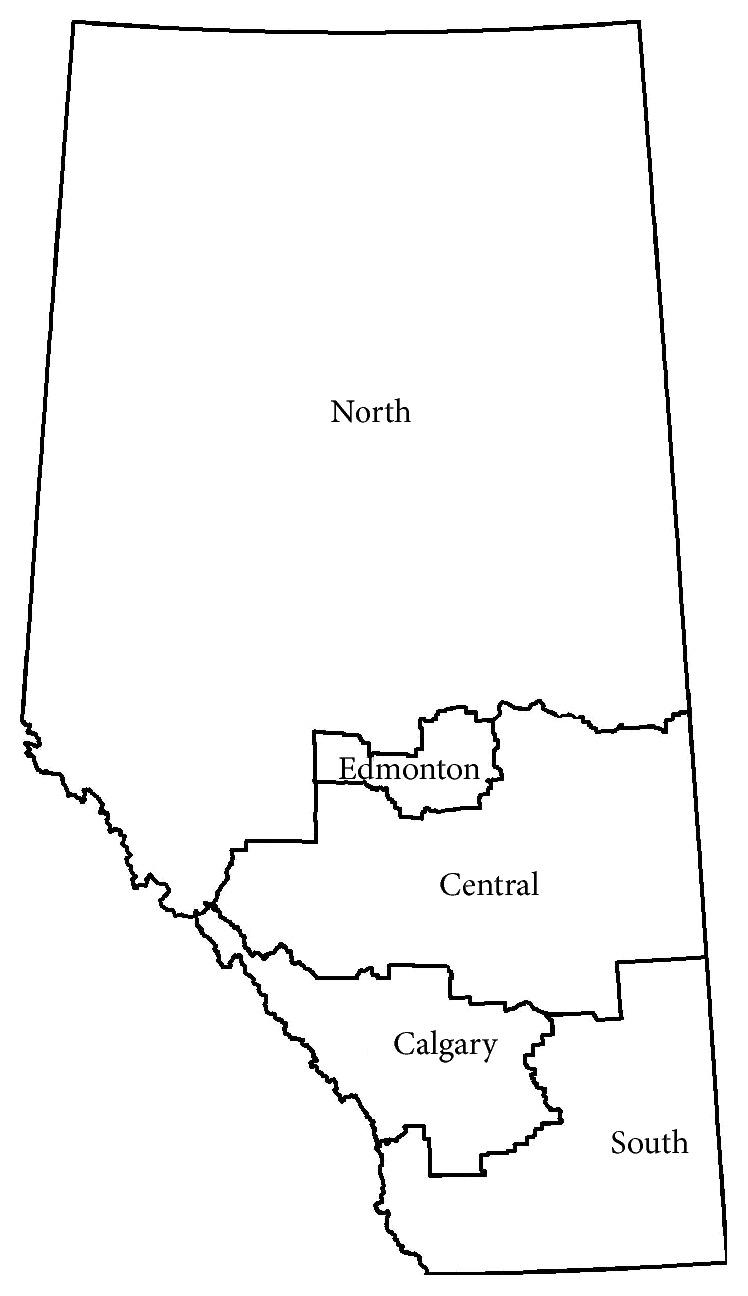
Map of Alberta depicting administrative health zones.

Two age groups were formed with patients classified as either 55–64 or 65+ based on age at time of ED visit. Alberta is comprised of five geographical and administrative health zones (North, Edmonton, Central, Calgary, and South; Figure 1) that are responsible for the delivery of health care. The AHCIP records the zone of residence of registrants at the end of the fiscal year.

### 2.4. Data Analysis

For each month, COPD ED visit rates per 100,000 individuals were calculated by age group, sex, zone, and overall. Additionally, rates for age group within each sex were calculated. Since population counts were only available at the end of each fiscal year, linear interpolation and extrapolation were used to obtain monthly population estimates and the months were normalized to 30 days so that comparisons were made across equal time periods. Patient demographics were summarized using frequencies and percentages, and the overall rates during the period were summarized by the average monthly COPD ED visits per 100,000 individuals. Three ED visits with missing zone were excluded from the zone specific analyses.

In order to summarize and differentiate between seasonal and trend components of the time series, seasonal-trend decomposition based on LOESS (STL) [12] was used. Seasonal-trend decomposition utilizes the nonparametric LOESS smoothing technique to decompose a time series into three distinct additive components: trend, seasonal, and remainder/residual. Because there was no obvious reason for the seasonal variation to differ across the years in this study, the seasonal components of the decomposed time series were held constant across years for each month (e.g., for a given series, the seasonal component for January does not vary across each year). For each subseries and overall, the percentage change in the trend component was calculated between the first and last months of the study (April, 1999, and March, 2011), and the highest and lowest seasonal months were compared.

To estimate parametric models for monthly rates of COPD ED visits over time, seasonal autoregressive integrated moving average (SARIMA) models were considered. SARIMA models can be denoted by ARIMA(*p*, *d*, *q*) × (*P*, *D*, *Q*)_*S*_ where *d* is the order of nonseasonal differencing, *p* and *q* are the order of nonseasonal autoregressive (AR) and moving average (MA) terms, respectively, *D* is the order of seasonal differencing, *P* and *Q* are the order of seasonal AR and MA terms, respectively, and *S* is the order of seasonality (e.g., 12 for monthly data) [13]. Additional details on these models are provided in the appendix. The order of seasonal and nonseasonal differencing needed to achieve stationarity of the series was determined using the OCSB test [14] and KPSS test [15], respectively, and validated by visual inspection of the autocorrelation function (ACF) and partial autocorrelation function (PACF) plots. A SARIMA model was then obtained by finding the parameters that yielded the smallest Akaike Information Criterion [16] (AIC) among all possible models. Residual plots were visually inspected to assess appropriateness of the final model. To evaluate potential forecasting ability of the models, the optimal SARIMA model for each time series was fit to the monthly data for the first 11 years of the study (April, 2000, to March, 2010), and forecasts were then generated for the next 12 months (April, 2010, to March, 2011). The predicted rates were plotted against the actual observed rates and the accuracy of the predictions was summarized using the mean absolute percent error (MAPE).

SAS version 9.4 for Linux (SAS Institute Inc., Cary, NC) and R version 3.2.0 for Windows (including the “stats” and “forecast” packages) were used to carry out all statistical analyses.

### 2.5. Ethics

The study was approved by the University of Alberta's Health Research Ethics Board and patients were not contacted during this study. The data are reported in aggregate, and small cell sizes are suppressed to protect anonymity.

## 3. Results

### 3.1. Patient Demographics, Trend, and Seasonality

There were a total of 188,824 ED visits for COPD between April, 1999, and March, 2011 (197.7 and 232.6 visits per 100,000 individuals, resp.), of which most (76.5%) listed COPD as the primary diagnosis. Average monthly rates of COPD ED visits per 100,000 individuals were higher for males than females (218.6 versus 180.2), higher for ages 65 and over compared to ages 55–64 (278.1 versus 109.5), and varied by geographic zones, ranging from a low of 108.6 in the Calgary zone to a high of 463.9 in the North zone (Table 1).

**Figure 2:**
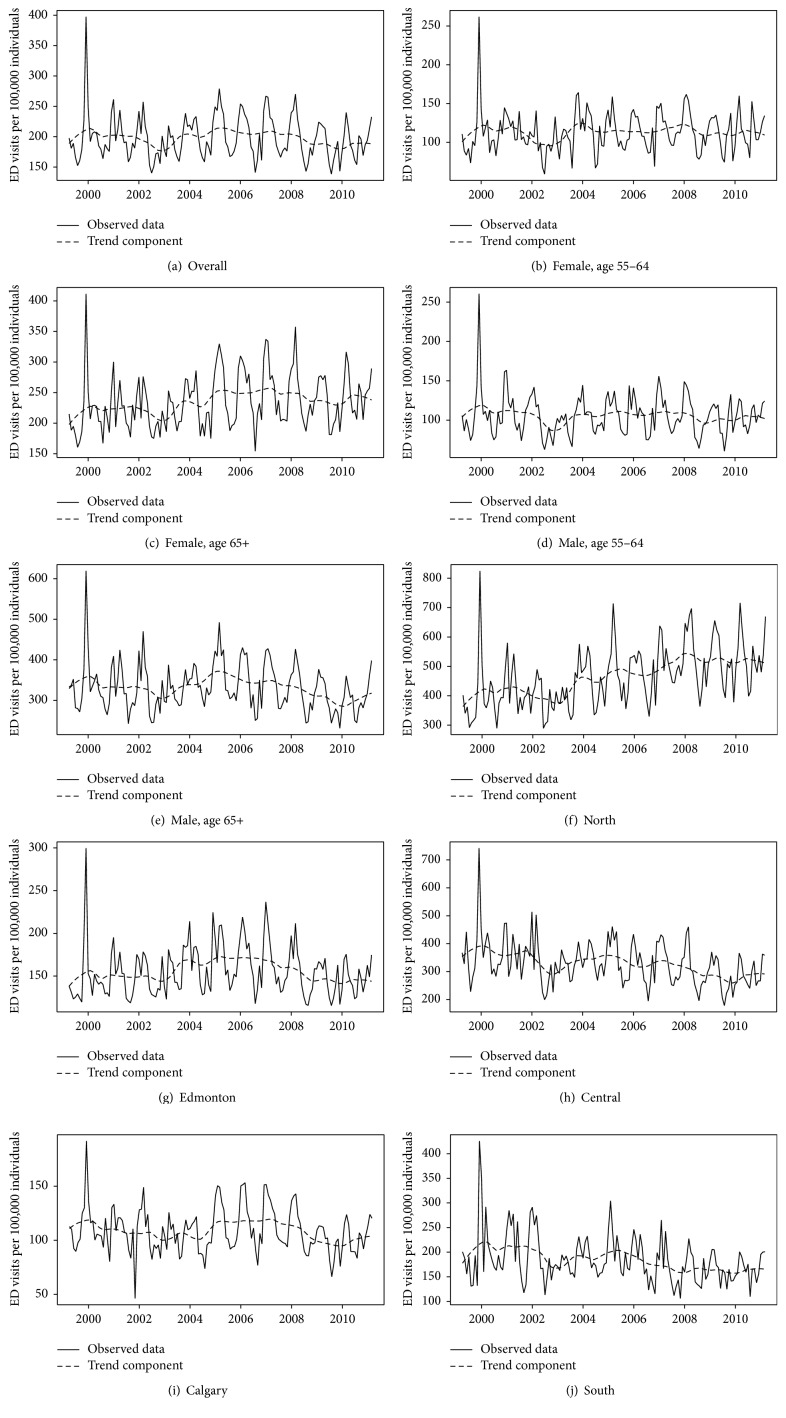
Monthly COPD ED visits per 100,000 individuals, April, 1999, to March, 2011, with overlay of trend component.

**Figure 3:**
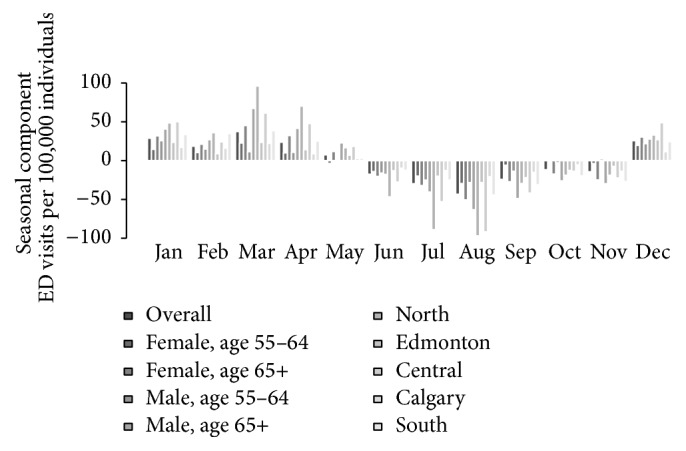
Comparison of seasonal components by month.

Each time series is shown in Figure 2, with the trend component obtained using STL superimposed to aid in visualization of the long term trend. The seasonal component of each series was plotted by month (Figure 3) and highlights the similarity in the seasonal component for each series. Of note, the overall series and all subgroups had the lowest rates in August and the highest rates typically in March (Table 1) and, in general, had higher rates in the winter and spring months (December to May) and lower rates in the summer and fall months (June to November) as is evident by the respective positive and negative seasonal (Sawtooth patterns) components in Figure 3.

Despite similar seasonality across each series, there existed different patterns of long term trend. The trend component for the overall rate of COPD ED visits was relatively flat, with a 0.4% decrease from April, 1999, to March, 2011. The differences in sex were small but opposite in direction (10.8% increase for females; 9.7% decrease for males). For females, the age 65+ group increased by 20.2% compared to only 8.6% for age 55–64, while for males both age groups saw small decreases (3.5% for age 65+ and 1.8% for age 55–64). Three of the zones saw only small changes over the time period, including the urban zones of Edmonton (4.1% increase) and Calgary (6.5% decrease), as well as the South (6.9% decrease). The Central zone, however, had a decrease of 17.6% and the North zone had an increase of 40.6% over the time period (Table 1).

### 3.2. Time Series Modeling

To parametrically model the ED visit rates for COPD over time, SARIMA models were fit for each series. The OCSB test suggested seasonal differencing was not required for any of the series, and the KPSS test suggested nonseasonal differencing was required only for the North, Central, and South zones to achieve stationarity. For the overall series, the model that resulted in the smallest AIC was ARIMA(1, 0, 0) × (1, 0, 1)_12_, with AIC 1337.9, although several models had similar values of AIC suggesting other similar models are likely equally appropriate. The resulting optimal models for each series are shown in Tables 2 and 3.

**Figure 4:**
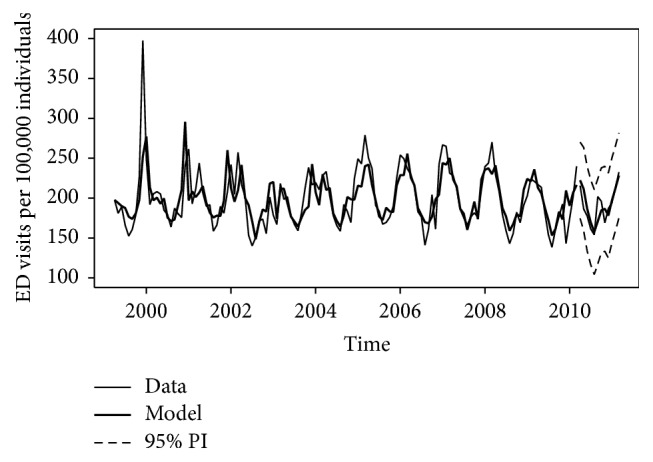
Comparison of observed and predicted rates based on the first 11 years of data.

To evaluate the predictive ability of the optimal model for the overall series, the model parameters were estimated using the first 11 years of monthly data (April, 1999, to March, 2010; 132 monthly values) and then used to predict the last year of our period (April, 2010, to March, 2011; 12 monthly values), including 95% prediction intervals. The estimated model performed well, with the 95% prediction interval containing the actual values for all of the 12 time points (Figure 4). The MAPE for the overall model was 5.7% and ranged from 6.4% to 11.3% for each of the subseries.

## 4. Discussion

We described the monthly rates of presentations to the ED for COPD during 12 years in a Canadian province and used time series modeling techniques to quantify the patterns. With over 188,000 ED visits for COPD during the study period, we demonstrated stable presentation rates, variations across age, sex, and location, and a consistent pattern reflecting the influences of environmental conditions. These findings were similar to our earlier investigation of ED visits for COPD in Alberta during 1999 to 2005 [4].

The stable presentation rates observed in this study are similar to those seen in other jurisdictions, where stable temporal trends were observed during 1999 to 2010 (adults aged ≥25 years) [17] or 2001 to 2012 (adults aged ≥18 years) [18]. We observed seasonal highs and lows in late winter and summer, respectively, and the seasonal components for ED presentations for COPD are similar to those seen with asthma during the same time period [19]. High circulating respiratory pathogens, air recirculation, and indoor restriction in activities in this northern region contribute to the patterns seen epidemiologically. Emergency department presentations for asthma, however, have demonstrated a dramatic and consistent decline over the same period and the COPD data showed a decrease for only one geographic area of the province. Further, there was evidence of a decreasing trend for males and the age-adjusted rate in 2010 was lower for men than women in the United States [17].

Relatively few studies have used time series analysis methods to investigate patterns of COPD presentations. In Australia, COPD mortality rates during 1922–2005 were used to create functional time series models and forecast age- and sex-specific mortality rates for 2006 to 2025 [20]. Multivariate time series methods have been used to model weekly rates of respiratory disease hospitalization and viral isolations in Ontario, Canada, during 1997 to 2002 [21]. COPD, bronchiolitis, pneumonia, respiratory syncytial virus, and influenza were modeled together, requiring seasonal and autoregressive terms (order 2), and influenza was the primary influence of COPD admissions. COPD hospital admissions during 1972 to 1992 in Finland showed seasonality and increasing trends, and a seasonal ARIMA model was obtained [22]. These studies are generally descriptive analyses and do not necessarily provide estimates of the effect of explanatory factors on the time series. More generally, authors have used Poisson regression or other models that have a time component to investigate admission rates [3, 23] and diagnosis rates [24] after changes in care recommendations or policies for COPD, to estimate COPD incidence or prevalence [25], or to determine the effects of pollution on COPD ED presentations [26–30] and deaths [31].

The study has several strengths and limitations. The large sample size, 12-year time frame, the use of population-based databases, and time series methods are all strengths of the study. In addition, the use of diagnostic codes assigned by trained medical records nosologists strengthens the validity of the work.

Study limitations include the lack of linked data on treatment or contact with other health care services before the ED treatment; our focus on ED visits did not identify patients with COPD who used alternative sources for the delivery of acute care. In addition, the diagnosis of COPD is notoriously difficult to validate without supplemental records such as pulmonary function testing, simple imaging (e.g., chest radiography), or advanced imaging (e.g., CT scans of chest). While the diagnosis was not confirmed and this may both over- and underestimate COPD cases, ED-based research suggests that the diagnosis of COPD is valid and underestimates the problem [23]. Finally, any differences in the observed patterns may be because of differential access to emergency health service due to health disparities and not necessarily reflect systemic differences in illness distributions.

In summary, rates of presentations to the ED for COPD have been remarkably stable and demonstrate a consistent pattern reflecting the influences of a cold-weather environment. During the cold winter months, indoor exposure, seasonal viral infections, and use of enclosed spaces predispose patients, and especially those with COPD, to upper respiratory tract infections, influenza, and pneumonia complications. SARIMA models fit the overall and subgroup data well and subgroups have similar parameter estimates. Such models can be helpful for forecasting and future health care planning.

## Figures and Tables

**Table 1:** Patient demographics and time series characteristics.

	COPD ED visits, *n* (%)	Mean monthly COPD ED visits/100,000	Change in trend component, %	Seasonal high month	Seasonal low month
Overall	188,824	198.4	−0.4	March	August
Sex					
Female	90317 (47.8)	180.2	10.8	March	August
Age 55–64	25483 (28.2)	113.2	8.6	March	August
Age 65+	64834 (71.8)	234.5	20.2	March	August
Male	98507 (52.2)	218.6	−9.7	March	August
Age 55–64	24132 (24.5)	105.8	−1.8	January	August
Age 65+	74375 (75.5)	331.7	−3.5	March	August
Zone					
North	45411 (24.0)	463.9	40.6	March	August
Edmonton	48314 (25.6)	155.6	4.1	December	August
Central	44111 (23.4)	328.7	−17.6	March	August
Calgary	35160 (18.6)	108.6	−6.5	March	August
South	15825 (8.4)	184.3	−6.9	March	August

**Table 2:** Overall optimal model and parameter estimates for COPD ED visit rates.

Parameter	Overall
Optimal model	(1,0, 0) × (1,0, 1)_12_
Intercept	197.31 (10.31)
AR(1)	0.44 (0.08)
MA(1)	—
MA(2)	—
Seasonal AR(1)	0.89 (0.07)
Seasonal AR(2)	
Seasonal MA(1)	−0.52 (0.16)
Seasonal MA(2)	—
AIC	1337.9

Parameter estimates are displayed with standard error in parentheses.

**Table 3:** Subseries optimal models and parameter estimates for COPD ED visit rates.

Parameter	Female, age 55–64	Female, age 65+	Male, age 55–64	Male, age 65+	North	Edmonton	Central	Calgary	South
Optimal model	(0,0, 1) × (2,0, 0)_12_	(0,1, 2) × (2,0, 0)_12_	(1,0, 0) × (2,0, 0)_12_	(1,0, 0) × (1,0, 1)_12_	(0,1, 2) × (1,0, 1)_12_	(0,0, 1) × (1,0, 1)_12_	(1,1, 1) × (1,0, 1)_12_	(1,0, 0) × (1,0, 2)_12_	(1,1, 1) × (1,0, 1)_12_
Intercept	113.49 (4.48)	—	106.99 (5.97)	328.80 (16.10)	—	152.99 (6.16)	—	108.63 (5.65)	—
AR(1)	—	—	0.39 (0.08)	0.46 (0.08)	—	—	0.32 (0.09)	0.46 (0.08)	0.22 (0.09)
MA(1)	0.33 (0.07)	−0.61 (0.08)	—	—	−0.60 (0.08)	0.42 (0.08)	−0.97 (0.02)	—	−0.97 (0.02)
MA(2)	—	−0.37 (0.08)	—	—	−0.34 (0.07)	—	—	—	—
Seasonal AR(1)	0.32 (0.09)	0.38 (0.09)	0.32 (0.09)	0.88 (0.07)	0.92 (0.07)	0.83 (0.08)	0.92 (0.06)	0.95 (0.06)	0.93 (0.06)
Seasonal AR(2)	0.19 (0.10)	0.31 (0.09)	0.33 (0.10)	—	—	—	—	—	—
Seasonal MA(1)	—	—	—	−0.56 (0.16)	−0.67 (0.17)	−0.46 (0.14)	−0.68 (0.13)	−0.54 (0.13)	−0.71 (0.14)
Seasonal MA(2)	—	—	—	—	—	—	—	−0.23 (0.12)	—
AIC	1309.7	1404.7	1269.8	1482.2	1635.5	1303.8	1590.1	1185.8	1467.7

Parameter estimates are displayed with standard error in parentheses.

## Appendix

We provide a brief overview of the Box et al. [13] time series modeling techniques. A more extensive discussion and additional details can be found elsewhere [32].

Suppose that *Y*_*t*_ is the outcome of interest at time *t* (in our analysis, *Y*_*t*_ is the COPD visit rate for month *t*, where *t* = 1 [April, 1999] to *t* = 132 [March, 2011]). Let *ε*_*t*_ denote the random error at time *t* and let *B* denote the lag operator (i.e., *BY*_*t*_ = *Y*_*t*−1_). A general autoregressive (AR) moving average (MA) model can be denoted by ARMA(*p*, *q*) and specified as Φ(*B*)*Y*_*t*_ = Θ(*B*)*ε*_*t*_ where Φ(*B*) and Θ(*B*) are polynomials in *B* of order *p* and *q*, respectively. For example, if Φ(*B*) = 1 − Φ_1_ *B* − Φ_2_ *B* ^2^ and Θ(*B*) = 1 − *θ*_1_ *B* then the resulting ARMA(2, 1) model can be written as *Y*_*t*_ − Φ_1_ *Y*_*t*−1_ − Φ_2_ *Y*_*t*−2_ = *ε*_*t*_ − *θ*_1_ *ε*_*t*−1_, and with some rearrangement the model becomes *Y*_*t*_ = Φ_1_ *Y*_*t*−1_ + Φ_2_ *Y*_*t*−2_ + *ε*_*t*_ − *θ*_1_ *ε*_*t*−1_. A seasonal ARMA model can be similarly represented. For example, if the season is denoted by *S*, then SARMA(*p*, *q*) × (*P*, *Q*)_*S*_ can be written as Φ_*S*_(*B*)Φ(*B*)*Y*_*t*_ = Θ_*S*_(*B*)Θ(*B*)*ε*_*t*_ where Φ_*S*_(*B*) and Θ_*S*_(*B*) are polynomials in *B* ^*S*^ of order *P* and *Q*, respectively. For example, if *S* = 12 and *P* = 2, the polynomial would be specified as Φ_12_(*B*) = 1 − Φ_12,1_ *B* ^12^ − Φ_12,2_(*B* ^12^)^2^.

To enable the time series analysis approach, the series must not exhibit trends in the mean or variance. If a series does exhibit trends, the series can be differenced. Let the difference operator be denoted by Δ (Δ = 1 − *B*). For example, the ARMA(*p*, *q*) model can be fit on the series *Z*_*t*_ = Δ*Y*_*t*_ = *Y*_*t*_ − *Y*_*t*−1_, rather than on *Y*_*t*_, to yield an autoregressive integrated moving average model, ARIMA(*p*, *d*, *q*), where *d* denotes the order of differencing (*d* = 1 in this example). Further, a seasonal difference may be required and the modeling is based on *Z*_*t*_ = (Δ^*S*^)^2^ *Y*_*t*_ = (1 − *B* ^*S*^)^2^ *Y*_*t*_, for example. In this case, a SARIMA(*p*, *d*, *q*) × (*P*, *D*, *Q*)_*S*_ would denote a model with a seasonal difference of order *D* (*D* = 2 in this example).

In general, the analyst would perform an iterative procedure to determine a suitable model. The autocorrelation function (ACF) and partial autocorrelation function (PACF) would be plotted to identify the preliminary values for *d* and for *D* and *S* if required. The series would be differenced according to these settings and the ACF and PACF would be examined on the differenced series to identify tentative choices for *p* and *q* and *P*, *Q*, and *S* if required. This step is referred to as the model identification step. The model would be fit using these choices to obtain the parameter estimates (e.g., Φ_1_). The residuals from the model fit should follow “white noise” and a good fit will not exhibit patterns in the residuals. If patterns are seen, the analyst should reconsider the choices made in the model identification step and provide other options for *p* and *q* (and *P* and *Q*) to provide a model that fits the data more appropriately.
